# Transcriptome analysis of effects of *Tecrl* deficiency on cardiometabolic and calcium regulation in cardiac tissue

**DOI:** 10.1515/med-2023-0880

**Published:** 2024-01-23

**Authors:** Shujia Lin, Shun Chen, Qiuping Lin, Tingting Xiao, Cuilan Hou, Lijian Xie

**Affiliations:** Department of Cardiology, Shanghai Children’s Hospital, School of Medicine, Shanghai Jiaotong University, Shanghai, 200062, China; Department of Pediatrics, Jinshan Hospital, Fudan University, Shanghai, 201508, China; NHC Key Laboratory of Medical Embryogenesis and Developmental Molecular Biology, Shanghai Key Laboratory of Embryo and Reproduction Engineering, Shanghai, 200040, China

**Keywords:** catecholaminergic polymorphic ventricular tachycardia, Tecrl, RNA-seq, WGCNA

## Abstract

Catecholaminergic polymorphic ventricular tachycardia (CPVT) is a hereditary heart disease characterized by bidirectional or polymorphic ventricular tachycardia and an increased risk of sudden cardiac death. Although trans-2,3-enoyl-CoA reductase like (*TECRL*) is a newly reported pathogenic gene leading to CPVT that can influence intracellular calcium regulation, the unidentified mechanism underlying the pathogenesis of TECRL deficiency-mediated CPVT remains mainly elusive. In the present study, *Tecrl* knockout (KO) mice were established and the differentially expressed genes (DEGs) were investigated by RNA-sequencing from the heart tissues. In addition, 857 DEGs were identified in *Tecrl* KO mice. Subsequently, a weighted gene co-expression network analysis was conducted to discern the pivotal pathways implicated in the *Tecrl*-mediated regulatory network. Moreover, pathway mapping analyses demonstrated that essential metabolism-related pathways were significantly enriched, notably the fatty acid metabolic process and calcium regulation. Collectively, the data suggested a synergistic relationship between *Tecrl* deficiency and cardiometabolic and calcium regulation during the development of CPVT. Therefore, further studies on the potential function of TECRL in cardiac tissues would be beneficial to elucidate the pathogenesis of CPVT.

## 1 Introduction

Sudden death disease resulting from arrhythmia is one of the leading causes of mortality in patients without cardiac structural alterations. Catecholaminergic polymorphic ventricular tachycardia (CPVT) is an inherited ion channel disease that often occurs in prepuberal children (7–12 years old), with an incidence of 1/10,000 [1]. Its prominent clinical feature is bidirectional or polymorphic ventricular tachycardia caused by physical exercise or emotional stress and its first clinical manifestation is syncope or sudden death. CPVT is usually divided into several subtypes, including CPVT1, CPVT2, and CPVT3 and other subtypes [2]. Among them, CPVT1, which accounts for 50–60%, is one of the most common autosomal dominant forms caused by ryanodine receptor 2 (*RYR2*) mutation. CPVT2, which accounts for 5%, is an autosomal recessive form caused due to calcequestrin 2 (*CASQ2*) mutation [3–5], and CPVT3, which accounts for 1%, is also an autosomal recessive form caused by trans-2,3-enoyl-CoA reductase like (*TECRL*) mutation [6]. Our group reported a unique heterozygosity mutation in *TECRL* gene sequence of a boy diagnosed with CPVT3 and successfully generated a brand new *Tecrl* knockout (KO) mouse model based on this basis [7,8]. However, the mechanism of CPVT3 caused by *TECRL* requires further investigation.

TECRL localizes to the endoplasmic reticulum (ER) [9]. Previous studies have reported that *TECRL* deficiency was accompanied by reduction in the expression levels of RYR2 and CASQ2, leading to aberrant regulation of [Ca^2+^]_*i*_ in human pluripotent stem cell-derived cardiomyocytes (hiPSCs-CMs) [6]. *TECRL* also has an important sequence identity with *TECR*, which participates in the elongation of very long-chain fatty acids [10]. Previous studies have shown that oxidation disorders of very long-chain fatty acid increase the incidence of delayed depolarizations (DADs) and diastolic [Ca^2+^]_*i*_, leading to the occurrence of arrhythmia [11]. As shown in our previous study, *TECRL* deficiency results in impaired mitochondrial function in both hearts and hiPSC-CMs [7]. In addition to the regulation of calcium processing, the potential of TECRL to interact with other pathways involved in the regulation of CPVT remains to be determined.

Recently, an increasing number of mouse models harboring CPVT-linked mutations have been established in *Ryr2* (R4496C, N2386I, A165D) and *Casq2* (D307H, DeltaE9/DeltaE9) [5,12–14]. In agreement with these findings, mouse genetic studies have suggested that these mutations are characterized by stress-induced arrhythmia with typical CPVT phenotypes notably abnormal calcium homeostasis and ultrastructural changes of mitochondria [5,12–14]. TECRL has been considered to be closely associated with [Ca^2+^]_*i*_, with the exception of animal models reported in the previous study conducted by our group [7]. In the past decade, the advancement of bioinformatic analyses utilizing high-throughput sequencing has significantly enhanced our comprehension of the underlying molecular mechanisms of CPVT through the identification of differentially expressed genes (DEGs). It is important to note that the diversity of algorithms utilized in bioinformatic analysis can heavily influence the results. The weighted gene co-expression network analysis (WGCNA) is a method that identifies gene modules with similar expression patterns and disease-related hub genes without the need for DEG analysis. Therefore, to further investigate the potential function of TECRL on cardiac myocytes, we performed RNA-seq on the hearts of *Tecrl* ^*−*/−^ mice followed by WGCNA. Herein, we reported that *Tecrl* deficiency created a reliance on fatty acid metabolism and Ca^2+^ handling. Therefore, TECRL may be useful as a therapeutic target in human CPVT.

## 2 Materials and methods

### 2.1 Animal model

Eight-week-old C57BL/6 mice and *Tecrl* KO mice were obtained from Shanghai Laboratory Animal Center. All mice had unrestricted access to water and food and were housed under 12 h dark–light conditions.


**Ethics statement:** All animal experiments were performed under licenses granted from the Ethics Committee of Experimental Research of Shanghai Children’s Hospital, School of Medicine, Shanghai Jiaotong University. This study was carried out in compliance with the Animal Research: Reporting of *In vivo* Experiments (ARRIVE) guidelines.

### 2.2 Western blotting

Heart tissues obtained from 8-week C57BL/6 mice were lysed in RIPA cell lysate buffer supplemented with protease (Roche Diagnostics) and phosphatase inhibitors (Roche Diagnostics); tissue lysates were subsequently centrifuged at 12,000 rpm for 10 min at 4°C. A bicinchoninic acid kit (Applygen Technologies, Inc.) was used to assess the protein concentration. Polyvinylidene fluoride membranes were incubated with the following primary antibodies overnight at 4°C: anti-TECRL (1:1,000; Aviva System Biology, Inc.) and anti-Tubulin (1:1,000; Cell Signaling Technology, Inc.). The secondary antibody (1:2,000; Cell Signaling Technology, Inc.) was diluted and incubated for 1 h at room temperature. An immunology scanner (GS-800, Bio-Rad Laboratories, Inc.) was used to measure the densities of the protein bands following exposure of the membranes to a chemiluminescent substrate (ECL, PerkinElmer, Inc.).

### 2.3 Sample collection and total RNA isolation

At 8 weeks of age, male and female mice were anesthetized with pentobarbital (100 mg/kg intraperitoneal dose) and subsequently sacrificed by cervical dislocation. Following verification of respiratory and cardiac arrest, animal death was confirmed. For subsequent processing, the heart tissues of each mouse were collected and stored at −80°C. Male and female mice were divided into a wild-type (WT) group and a *Tecrl* ^*−*/−^ group. Each group contained three mice. TRIzol^®^ (Invitrogen; Thermo Fisher Scientific, Inc.) was added to the heart tissues of each group. Following sonication for three times (15 s each time), isopropyl alcohol was added to the mixture, which was allowed to stand for 10 min at room temperature. Subsequently, the mixture was centrifuged at 12,000 rpm for 10 min at 4°C. The supernatant was collected and washed with 75% alcohol. The final sample was stored at −80°C. RNA purity was assessed using the NanoPhotometer^®^ spectrophotometer (IMPLEN). The RNA concentration was measured using Qubit^®^ RNA Assay Kit in Qubit^®^ 2.0 Fluorometer (Thermo Fisher Scientific, Inc.).

### 2.4 Library preparation for transcriptome sequencing

RNA for each sample was used at 1.5 µg total amounts as input material for sample preparations. Random hexamer primers were used to synthesize the first strand cDNA and the second strand cDNA synthesis was synthesized using DNA PolymeraseⅠand RNase H. Finally, the library quality was assessed on the Agilent Bioanalyzer 2100 system. The RNA-seq data produced in the course of this investigation have been archived in the Gene Expression Omnibus (GEO) database and the corresponding accession number is GSE191112. The residual data can be found in the article, appendix, or source data file. The source data file is included with this manuscript.

### 2.5 Gene expression level quantification and differential expression analysis

The cDNA libraries from each mouse were sequenced. The transcript expression levels of each gene were calculated using fragments per kilobase of transcript per million mapped reads (FPKM). The DESeq2 R package (1.26.0) was used to perform differential expression analysis. *P* < 0.05 and |log_2_(FoldChange)| ≥0.58 were used to indicate a significant differential expression.

### 2.6 DEGs identification and functional enrichment analysis

The cDNA libraries from each mouse were sequenced. The transcript expression level of each gene was calculated using FPKM. The DESeq2 R package (1.26.0) was used to perform differential expression analysis. A *P* value <0.05 and |log2(FoldChange)| ≥0.58 were set as the thresholds for significant differential expression. For functional annotation and classification, DEGs were enriched to the gene ontology (GO) terms based on the DAVID database. GO terms with *P* values less than 0.05 were considered significantly enriched by DEGs. The enrichment of DEGs in the KEGG pathway was tested using KOBAS v3.0 software. KEGG terms with *P* values less than 0.05 were considered significantly enriched by DEGs.

### 2.7 WGCNA

The functional modules in RNA-seq data were clustered using the WGCNA package in R software, with a defined cut-off height of 0.25 for merging similar modules. The co-expression network was inputted with group phenotypes (*Tecrl* KO vs control, mice gender), and genes with high hub modularity were identified as hub genes in the modular–trait correlation analysis. A heatmap was utilized to visualize the correlation between gene modules and clinical traits, followed by the identification of modules associated with co-expression patterns (midnight blue module and royal blue module) and phenotypes. The appendix material contains the analysis results of both modules.

### 2.8 Real- time PCR

Total RNA was extracted from mice tissue using TRIzol^®^ reagent (Invitrogen; Thermo Fisher Scientific, Inc.). Complementary DNA was generated from 1,000 ng total RNA using oligo DT and SuperScript III reverse transcriptase (Invitrogen; Thermo Fisher Scientific, Inc.). Real-time PCR was performed using SYBR Green Master Mix (Roche Diagnostics) on a LightCycler 480 Instrument. The primers used are listed in Table A1.

### 2.9 Data collection from the GEO database

Transcription data of induced pluripotent stem cell-derived cardiomyocytes (iPSC-CMs) derived from patients diagnosed with Brugada Syndrome (BrS) were downloaded from the GEO database (GSE221945). According to the annotation information in the platform, the probes were converted to gene symbols. Four BrS samples (GSM6910336, GSM6910337, GSM6910338, and GSM6910339) and four control samples (GSM6910332, GSM6910333, GSM6910334, and GSM6910335) were identified in the GSE221945 dataset and were used in the present study. The data from GSE221945 were used to validate the function of Tecrl deficiency-related hub genes in fatty acid metabolism and their clinical implication in ventricular arrhythmia. The data procurement and application conformed to the GEO database principles and guidelines. The R packages “Pheatmap” was used to draw the heatmap.

### 2.10 Data analysis

Western blot parameters are presented as mean ± SEM. Data display was generated in GraphPad Prism 9.0 (GraphPad Software, Inc.). The DESeq2 R package (1.26.0) was used to analyze the differential expression analysis. For four-sample comparison in reverse transcription-quantitative PCR, one-way ANOVA was performed. *P* < 0.05 was considered to indicate the DEGs that demonstrated statistical significance.

## 3 Results

### 3.1 *Tecrl* expression in various tissues

**Figure 1:**
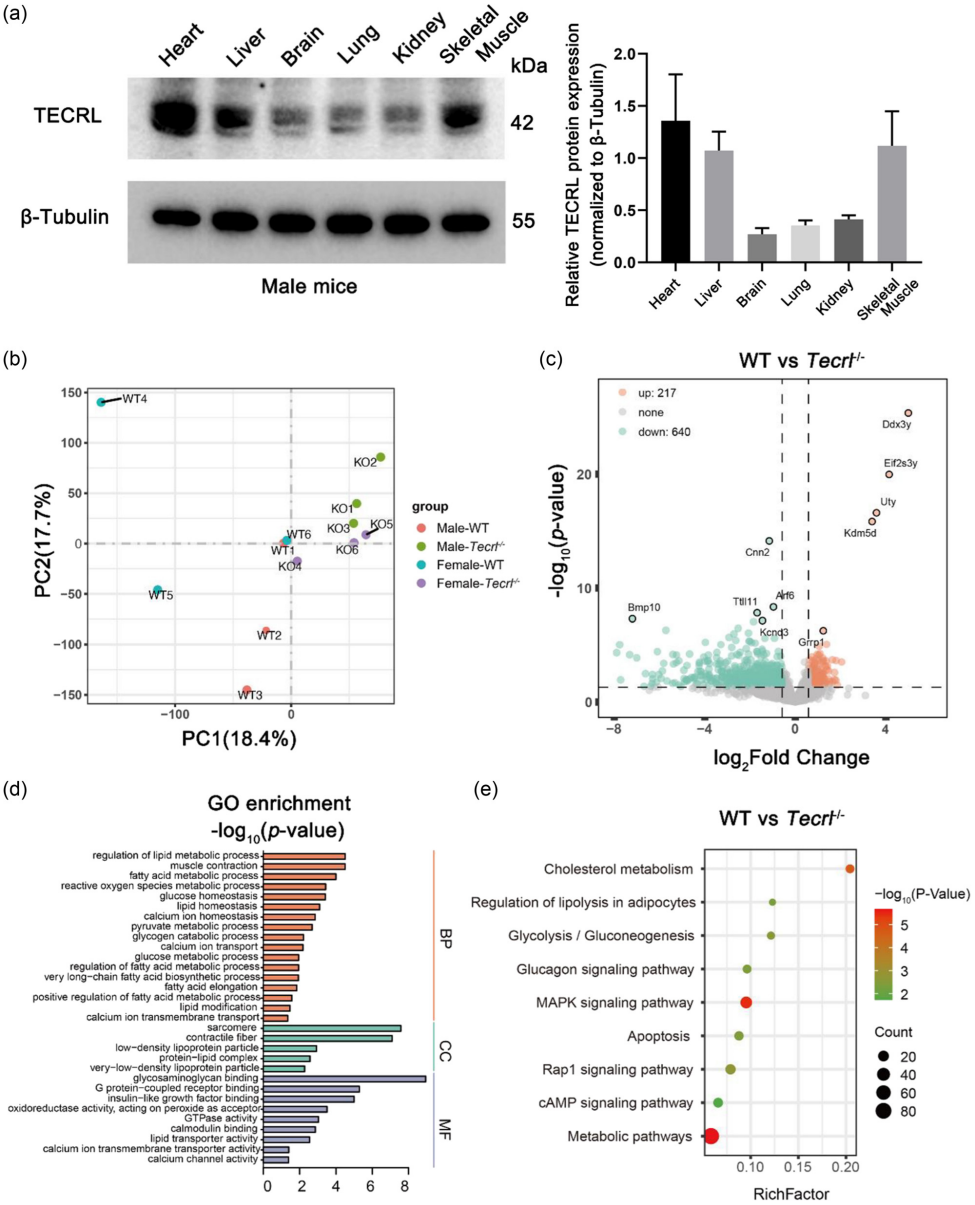
TECRL protein identification and functional enrichment analysis of DEGs. (a) Representative western blots of TECRL in different tissues of WT mice. (b) PCA plot of male and female mice samples. There were four groups, three mice in each group. An average of 5.28 ±  1.37 million raw reads per sample were obtained and PCA shows distinct patterns among groups. (c) Volcano plot of DEGs of male *Tecrl* ^*−*/−^ mice. Each point represents a single gene. Grey points represent the genes that have no significant changes, while the orange dots indicate the genes that are upregulated and green dots indicate the genes that are downregulated. (|log_2_(FoldChange)| ≥0.58, *P* value <0.05). (d) GO enriched terms associated with DEGs in male *Tecrl* ^*−*/−^ mice. (e) KEGG enriched pathways related to DEGs in male *Tecrl* ^*−*/−^ mice. BP, biological process; CC, cellular component; MF, molecular function.

In the previous study conducted by our group, *Tecrl* ^*−*/−^ mice were generated and the data indicated that *Tecrl* deficiency resulted in an abnormal electrocardiographic pattern and disrupted Ca^2+^ regulation [7]. To determine the tissue expression pattern of TECRL in WT mice, western blots were performed on multiple tissues of male mice. The protein expression analysis by western blots demonstrated that TECRL was ubiquitously expressed in heart, lung, kidney, brain, liver, and skeletal muscle (Figure 1a).

### 3.2 *Tecrl* deficiency effects on gene expression variation in male and female mice

To further investigate the impact of *Tecrl* deficiency on the mice, total RNA was extracted from the hearts of *Tecrl* ^*−*/−^ (male, *n* = 3; female, *n* = 3) and WT mice (male, *n* = 3; female, *n* = 3). The RNA samples were analyzed by RNA-seq. The transcript expression level of each gene was determined using FPKM. To visualize the pattern of distribution, a principal component analysis was conducted; its value was 40.9% with the first principal component (PC1) = 23.2%, and the second principal component (PC2) = 17.7% (Figure 1b). Deseq2 package in R was used to analyze the DEGs between the different groups. Finally, 640 (74.70%) downregulated and 217 (25.30%) upregulated genes were identified in the *Tecrl* ^*−*/−^ male mice (Figure 1c).

### 3.3 GO analysis of *Tecrl* deficiency in mice

The identified DEGs were categorized into biological processes (BP), cellular components (CC), and molecular functions. The BP of GO analysis revealed that the majority of DEGs were mainly enriched in the pathways related to calcium regulation and metabolism, regulation of lipid metabolic process, muscle contraction, fatty acid metabolism process, glucose homeostasis, lipid homeostasis, calcium ion homeostasis, pyruvate metabolic process, glycogen catabolic process, and calcium ion transport. The CCs mainly consisted of the sarcomere, contractile fiber, low-density lipoprotein particles, protein–lipid complex, and very-low-density lipoprotein particles (Figure 1d).

### 3.4 Functional enrichment analysis of *Tecrl* deficiency in mice

**Figure 2:**
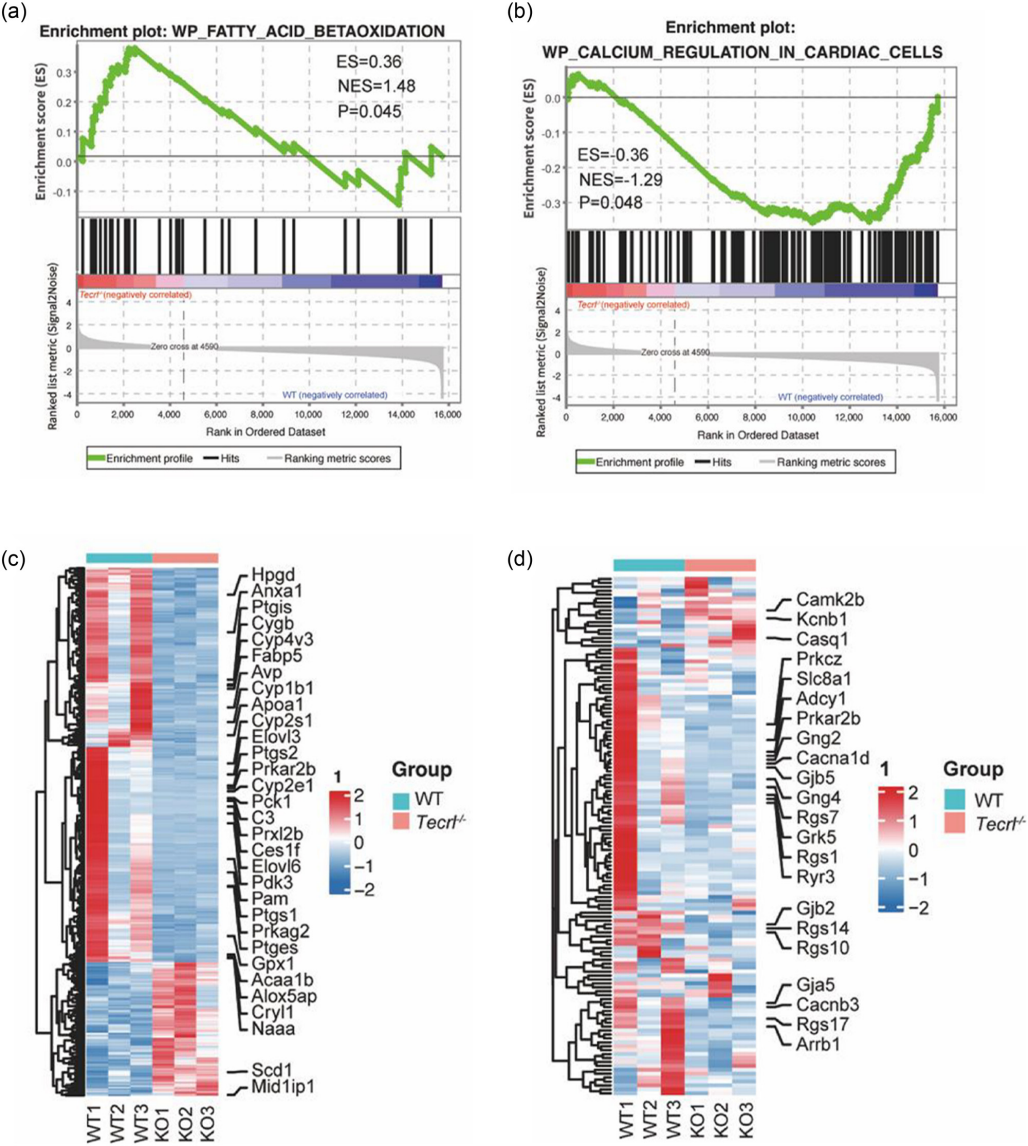
Hub gene selection and enrichment analysis: (a) GESA analysis of fatty acid beta-oxidation pathway, (b) GESA analysis of calcium regulation of cardiac cells pathway, (c) heatmap of fatty acid metabolism related genes in *Tecrl* ^*−*/−^ samples, and (d) heatmap of calcium handling related genes in *Tecrl* ^*−*/−^ samples.

KOBAS revealed the main pathways in which differentially expressed RNAs were enriched. In the male groups, KEGG analysis revealed the DEGs that were significantly enriched in 99 pathways. Among the pathways identified in the male *Tecrl* ^*−*/−^ group, metabolism-related pathways exhibited the highest number of DEGs, which included the metabolic pathway (86 genes), cholesterol metabolism (10 genes), regulation of lipolysis in adipocytes (7 genes), glycolysis/gluconeogenesis (8 genes), the glucagon signaling pathway (10 genes), the MAPK signaling pathway (28 genes), and the cyclic AMP signaling pathway (14 genes), most of which were related to fatty acid metabolism (Figure 1e). Gene set enriched analysis was also used to identify whether fatty acid beta-oxidation and calcium regulation of cardiac cells were related to *Tecrl* deficiency (Figure 2a and b). Subsequently, the expression levels of genes involved in fatty acid metabolism were shown by heatmap analysis (Figure 2c and d).

### 3.5 Pivotal gene modules identification through WGCNA

**Figure 3:**
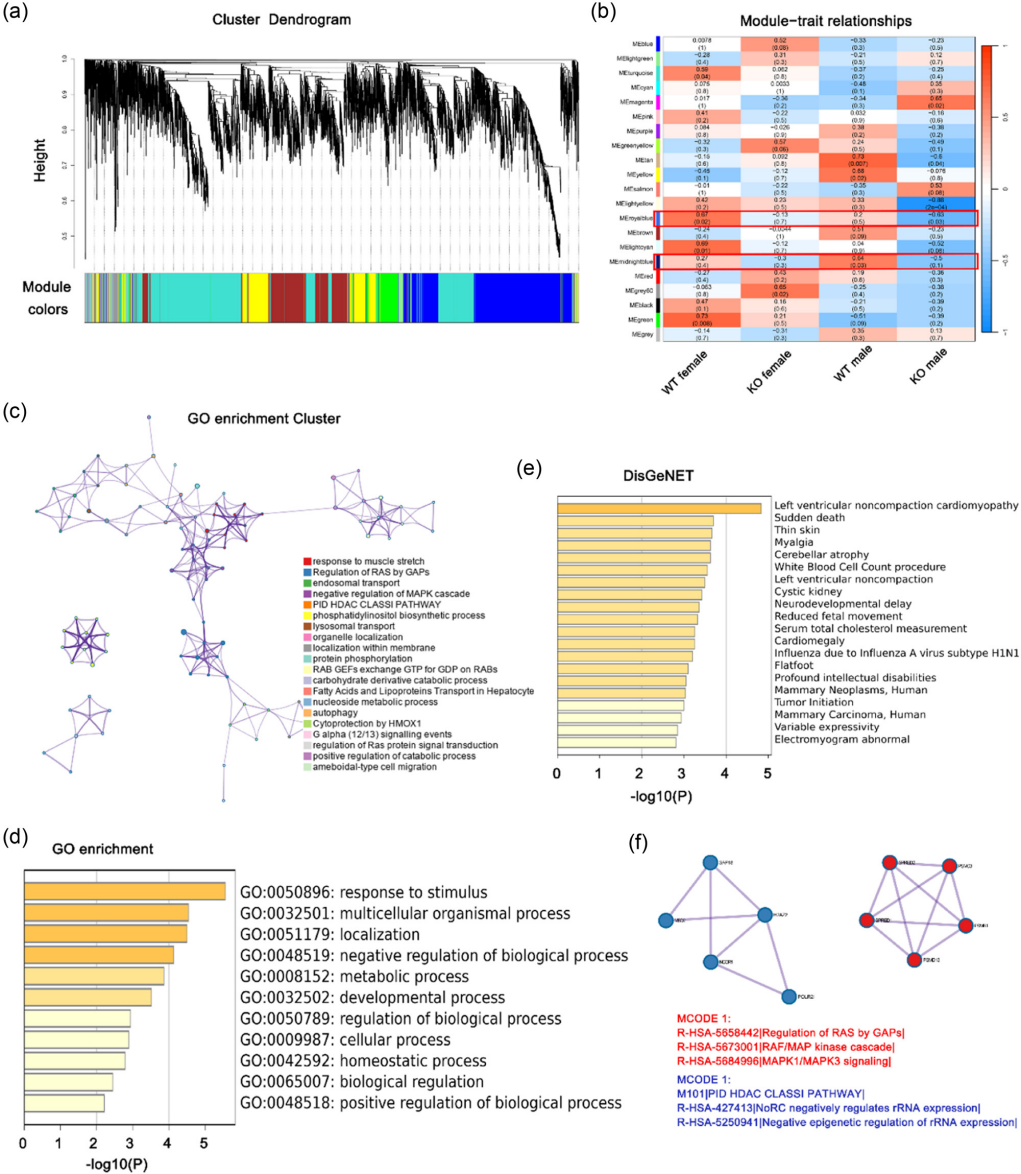
WGCNA revealing gene co-expression networks. (a) Utilizing WGCNA analysis, the dendrogram depicted the clusters of genes that were differentially expressed, based on various metrics. Each branch of the dendrogram represented an individual gene, while the colors beneath the branches represented a co-expression module. (b) The heatmap illustrated the correlation between gene modules and group phenotypes. The correlation coefficient within each cube indicated the degree of correlation between gene modules and traits, with a decreasing gradient from red to blue. (c) The functional enrichment analysis was conducted on *Tecrl* KO-related hub genes from the midnight blue and royal blue modules. (d) The DisGeNET terms of hub genes were enriched. (e) The hub genes underwent GO enrichment. The horizontal axis depicts the *P*-value of GO terms on Metascape using default parameters. (f) The top Molecular Complex Detection algorithm (MCODE) terms of hub genes related to *Tecrl* KO were identified. A network was formed by the protein–protein interactions (PPI) among *Tecrl* KO-related hub genes from the red module. The MCODE was utilized to detect the connected network components.

To methodically elucidate crucial gene modules and potential mechanisms within the cardiac tissues of *Tecrl* KO or control mice of both genders, a WGCNA was performed. A hierarchical clustering approach was employed to generate numerous randomly color-coded modules for the cluster dendrogram (Figure 3a). The modular–trait correlation heatmap is presented in Figure 3b. The present study reveals a consistent negative correlation between the *Tecrl* KO trait and the midnight blue module as well as the royal blue module in mice heart tissue. This suggests that the deficiency of *Tecrl* results in a downregulated expression of genes associated with these functional modules. Notably, the midnight blue module comprises 74 genes, including *CAV3*, *Rb1cc1*, and *Ryr2*, while the royal blue module consists of 44 genes, including *Psmc3*, *Psmd13*, and *Gsta4*, all of which are hub genes. The shared pathways of these two modules were identified via Metascape (http://metascape.org/). The top GO terms of the hub genes included the following: response to muscle stretch, fatty acid and lipoprotein transport in hepatocytes, carbohydrate derivative catabolic process, and other metabolism-related pathways; the findings suggested the significant alteration of the metabolic process following *Tecrl* deficiency (Figure 3c and d). An analysis of DisNET (Figure 3e) indicated the participation of cardiovascular dysfunction, specifically left ventricular noncompaction cardiomyopathy, cardiomegaly, and electromyogram abnormality. Additionally, the MCODE analysis demonstrated an enrichment in the regulation of RAS by GAPs, RAF/MAP kinase cascade, negative epigenetic regulation of rRNA expression, and other pathways (Figure 3f). Collectively, the data indicated that the hub genes exhibiting a negative correlation with KO of *Tecrl* expression in heart tissues may significantly impact the metabolic process, particularly the fatty acid metabolic process, in the context of cardiovascular dysfunction.

### 3.6 *Tecrl* deficiency effects on fatty acid metabolism in ventricular arrhythmia

**Figure 4:**
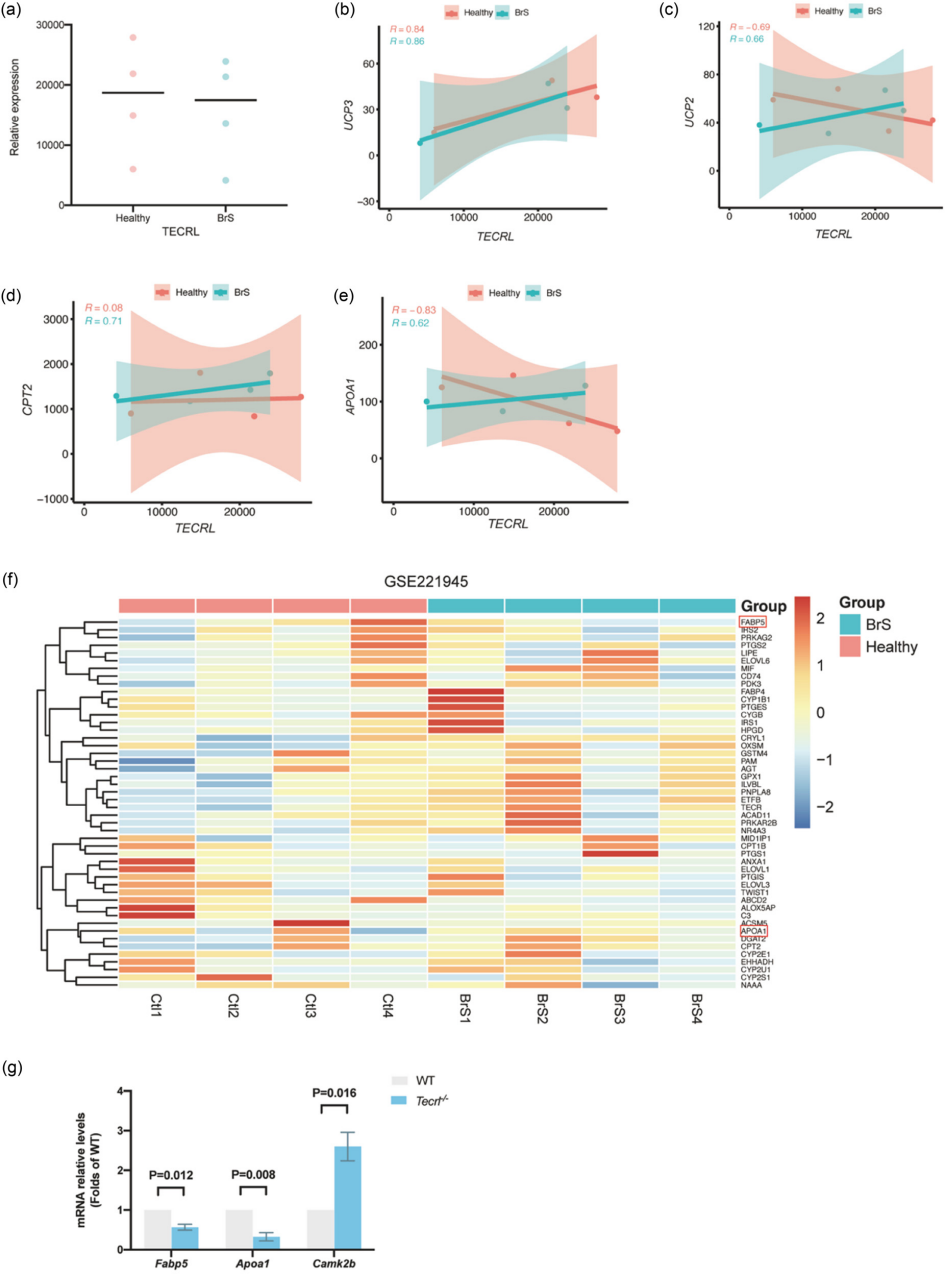
Tecrl deletion leads to significant representative changes. (a) TECRL expression in healthy and BrS patients. (b–e) The graphs of healthy group and Brs group showing a positive correlation of TECRL expression and (b) UCP3, (c) UCP2, (d) CPT2, and (e) APOA1. (f) The heatmap of 48 fatty acid metabolism related genes in BrS. (g) Changes in the mRNA level of Apoa1, Fabp5, and Camk2b.

To further explore the role of *TECRL* deficiency in the fatty acid metabolism and clinical implication in ventricular arrhythmia, the transcription data of iPSC-CMs were analyzed; iPSC-CMs were derived from patients diagnosed with BrS downloaded from GEO (GSE221945). TECRL was expressed at low levels in BrS samples (Figure 4a). Since fatty acid metabolism may play a crucial role in the pathogenesis of *Tecrl* ^*−*/−^ mice, the correlation of TECRL expression was analyzed with the key components (*APOA1*, *CPT2*, *UCP2*, *UCP3*) involved in the fatty acid metabolic pathways in BrS. In healthy iPSC-CMs, only UCP3 correlated positively with TECRL (Figure 4b). However, in iPSC-CMs derived from patients with BrS, all four genes were positively correlated with TECRL, which indicated the effects of TECRL on the fatty acid metabolism in patients with ventricular arrhythmia (Figure 4b–e). The transcription data also suggested that apolipoprotein A1 (APOA1) and fatty acid binding protein 5 (FABP5) exhibited a tendency to decrease in BrS, which was consistent with the results obtained from RNA-seq analysis (Figure 4f).

In the present study, qPCR experiments were performed in WT and *Tecrl* ^*−*/−^ mice. The mRNA levels of *Fabp5* and *Apoa1* were significantly decreased in *Tecrl* ^*−*/−^ mice (Figure 4g). To assess the relationship of arrhythmia to calcium handling, qPCR was performed to determine the mRNA expression levels of *Camk2b* in male *Tecrl* ^*−*/−^ mice. The results revealed upregulation of *Camk2b* expression in *Tecrl* ^*−*/−^ mice (Figure 4g). In summary, these results indicated that *TECRL* deficiency was positively correlated to the fatty acid metabolism pathway and played a role in Ca^2+^ transport.

## 4 Discussion

Given the elusive pathogenic characteristics of CPVT, it is imperative to comprehend the potential mechanisms and identify innovative therapeutic targets for this ailment. It is established that mutation of the critical pathogenic gene *TECRL* could lead to CPVT, which is associated with Ca^2+^ processing and the development of arrhythmia. Our present *in vivo* transgenic mouse model confirmed that the progression of CPTV derived from *Tecrl* deficiency is caused by alteration of fatty acid metabolism and Ca^2+^ processing. Further investigation into the mechanism of TECRL-regulated fatty acid metabolism and the Ca^2+^ metabolic processing will deepen the understanding of TECRL-induced CPVT.

In our work, 857 DEGs were identified in male *Tecrl* ^*−*/−^ mice using RNA-seq analysis. An upregulation in the expression levels of *Camk2b* was noted. GO analysis revealed that DEGs in male and female *Tecrl* ^*−*/−^ mice were significantly recruited in calcium handling-related pathways such as calcium ion homeostasis and calcium ion transport. Moreover, WGCNA is widely regarded as a superior approach for identifying internal functional modules among key genes. Despite this, there have been few studies utilizing WGCNA to elucidate the underlying mechanisms of CPVT. RNA-seq analysis was used to identify two crucial gene modules that exhibited a consistent negative correlation with the *Tecrl* KO trait in mouse heart tissues. GO enrichment analysis of these modules revealed significant alterations in metabolic processes following *Tecrl* KO, indicating that a deficiency in *Tecrl* led to the downregulation of metabolism-related pathways, ultimately resulting in cardiovascular dysfunction. The top two enriched pathways related to the ion channel in *Tecrl* ^*−*/−^ mice were the MAPK signaling and the Ras-proximate-1 signaling pathway, suggesting that CPVT may be associated with dysfunction of the ion channel, notably the processing of Ca^2+^. Calcium/calmodulin-dependent protein kinase II (CaMKII) is one of the isoforms involved in the regulation of intracellular calcium levels [15,16], which plays a pivotal role in the release of Ca^2+^ from the ER and abnormal sarcoplasmic reticulum (SR) Ca^2+^ leakiness [17,18]. Inhibition of CaMKII normalized the CPVT phenotype including Ca^2+^ handling and electrocardiogram (ECG), which indicated that CaMKII played a key role in the incidence of CPVT [17]. In addition, CPVT is a channelopathy caused by the unregulated pathological calcium release [19] in parallel with a high spontaneous Ca^2+^ release and Ca^2+^ waves [20], which is consistent with the results presented in the current study. More importantly, Devalla’s group previously revealed that mutation in TECRL in hiPSC-CMs led to abnormalities in intracellular calcium ([Ca^2+^]_*i*_) transients and increased susceptibility to trigger activity [6]. Our previous study also found that *TECRL* KO caused the alterations in calcium handling, which indicated that abnormal calcium handling may underly the mechanism by which *TECRL* deficiency induces CPVT (C. Hou and T. Xiao, unpublished data).

Another previous study reported that RYR2 may decrease glucose oxidation and increase glycolysis by inhibiting pyruvate dehydrogenase, as well as lowering Ca^2+^ signal amplitude and frequency [21]. Similarly, Santulli et al. found that the leaky RYR2 channels caused the impairment of insulin secretion, resulting in glucose intolerance [22]. Moreover, Li et al. expounded that BMAL1 regulates mitochondrial fission and mitophagy through mitochondrial protein BNIP3 and is critical in the development of dilated cardiomyopathy [23]. In our study, comparative analysis of differential gene expression showed that *Tecrl* deficiency was enriched in glucose metabolism, such as glucose metabolic process, glucose homeostasis, and glycogen catabolic process. Interestingly, Devalla et al. reported that *TECRL* deficiency was accompanied by a decrease in RYR2 in hiPSC-CMs [6], indicating that TECRL may have a potential effect on RYR2. Our previous study found that *TECRL* deficiency decreased the stability of RYR2, which suggested that *TECRL* deficiency may have an influence on RYR2. This suggested that TECRL played a key role in RYR2 expression, which may explain why DEGs were enriched in the glucose metabolism pathway.

Furthermore, we also observed that the most common pathway related to the DEGs was the fatty acid metabolism pathway in *Tecrl* ^*−*/−^ mice. And the TECRL was positively correlated with genes involved in the metabolism of fatty acids in BrS patients, which is a disorder also characterized by polymorphic ventricular tachycardia. Among the four genes, UCP2, UCP3, and CPT2 played a crucial role in fatty acid oxidation, it has proven that the downregulation of UCP2 and UCP3 impaired myocardial fatty acid oxidation and elevates the production of reactive oxygen species (ROS), subsequently increasing the incidence of arrhythmia [24,25]. We also identified the downregulation of *Apoa1* and *Fabp5* in male and female *Tecrl* ^*−*/−^ mice. Previous study revealed that *Apoa1* deficiency results in the abnormal calcium ion transport, while *Fabp5* deficiency increases oxidative stress [26,27]. Notably, *TECRL* has important individual sequences of TECR, which is a protein involved in the synthesis of fatty acids. Long chain fatty acids are responsible for the majority of energy for the normal human heart [28]. Impaired metabolism of fatty acids can contribute to the accumulation of the toxic lipids, which can in turn impair intracellular calcium handling and cardiac contractility [29]. Therefore, impaired metabolism of fatty acids is related to the occurrence of many cardiovascular diseases such as hypertrophic cardiomyopathy, diabetic cardiomyopathy, and arrhythmia [30–32]. Besides, previous study reported that very-long-fatty-acid deficiency may alter the ultrastructure of mitochondria and induce polymorphic ventricular tachycardia [33]. Priori’s group reported that mutation in *Ryr2* led to the increased DADs and mitochondrial abnormalities [34]. While increased mitochondrial ROS can stimulate the RYR leak and CaMKII, which is a critical mechanism of the incidence of CPVT [35]. Similarly, in our previous study, we found that *Tecrl* deficiency impaired the ultrastructure of mitochondria and increased mitochondrial ROS [7]. These indicate that the mechanism by which *TECRL* causes CPVT may be related to impaired fatty acid metabolism and ultrastructure of mitochondria.

Like all scientific research, our study had some limitations. For example, in sample selection, the lack of human heart tissue of CPVT for clinical validation is a major limitation in our study. Due to the preciousness of the cardiac tissue, we are currently unable to address this issue. In addition, a limited number of studies have been conducted utilizing WGCNA to elucidate the underlying mechanisms of CPVT. Although we have identified two crucial gene modules that exhibited a consistent negative correlation with the *Tecrl* KO trait in mouse heart tissues by RNA-seq analysis, it requires more validation in the real world. Hence, more work and further validation in multicenter, large-sample cohorts are urgently needed.

## 5 Conclusion

Our findings suggest a possible association between *Tecrl* deficiency-mediated CPVT and fatty acid metabolism as well as Ca^2+^ handling. This study provides a novel perspective on the function of TECRL and its role in CPVT. Further investigation into the protein function of TECRL may offer potential therapeutic targets for the prevention and treatment of CPVT in the future.

## Appendix

**Table A1:** Sequence of the primers used for validation

Gene name	Forward primer	Reverse primer
APOA1	5′-GTCCCAGTTTGAATCCTCCTCCTTG-3′	5′-AGGTTATCCCAGAAGTCCCGAGTC-3′
FABP5	5′-GCTAGGAGTAGGACTGGCTCTTAGG-3′	5′-TCTTCACTGTGCTCTCGGTTTTGAC-3′
CaMK2b	5′-CCCTGCCCATCTCCGACTCTC-3′	5′-AATGAGCTGCTCTGTGGTCTTGATG-3′
GAPDH	5′-ACCCAGAAGACTGTGGATGG-3′	5′-CACATTGGGGGTAGGAACAC-3′
